# The role of QRFP43 in the secretory activity of the gonadotrophic axis in female sheep

**DOI:** 10.1038/s41598-024-59801-1

**Published:** 2024-04-18

**Authors:** Bartosz Jarosław Przybył, Michał Szlis, Bartłomiej Wysoczański, Anna Wójcik-Gładysz

**Affiliations:** Department of Animal Physiology, The Kielanowski Institute of Animal Physiology and Nutrition, Polish Academy of Sciences, Instytucka 3, 05-110 Jabłonna, Poland

**Keywords:** QRFP43, Gonadotrophin-releasing hormone, Kisspeptin, Luteinising hormone, Follicle-stimulating hormone, Sheep, Reproductive biology, Neuronal physiology

## Abstract

In mammals reproduction is regulated by many factors, among others by the peptides belonging to the RFamide peptide family. However, the knowledge concerning on the impact of recently identified member of this family (QRFP43) on the modulation of the gonadotrophic axis activity is still not fully understood and current research results are ambiguous. In the present study we tested the in vivo effect of QRFP43 on the secretory activity of the gonadotrophic axis at the hypothalamic-pituitary level in Polish Merino sheep. The animals (n = 48) were randomly divided into three experimental groups: controls receiving an icv infusion of Ringer-Locke solution, group receiving icv infusion of QRFP43 at 10 μg per day and 50 μg per day. All sheep received four 50 min icv infusions at 30 min intervals, on each of three consecutive days. Hypothalamic and pituitaries were collected and secured for further immunohistochemical and molecular biological analysis. In addition, during the experiment a blood samples have been collected for subsequent RIA determinations. QRFP43 was found to downregulate Kiss mRNA expression in the MBH and reduce the level of IR material in ME. This resulted in a reduction of GnRH IR material in the ME. QRFP43 increased plasma FSH levels while decreasing LH levels. Our findings indicate that QRFP43 inhibits the activity of the gonadotropic axis in the ovine at the level of the hypothalamus and may represent another neuromodulator of reproductive processes in animals.

## Introduction

RF-amide peptides are a family of regulatory peptides possessing arginine and amidated phenylalanine (RF; Arg-Phe-NH2) sequence at their C-terminal. In mammals, some of these peptides have been characterised as major regulators of gonadotropin-releasing hormone (GnRH) neurons. In particular, two key RFamide peptides, kisspeptin (Kiss1) and gonadotropin-inhibitory hormone (GnIH), are emerging as the most important regulators of the reproductive axis. Some research has showed that Kiss1 may acts as the accelerator, whereas GnIH may plays a role as downregulator of GnRH neurons secretory activity of^1,2^.

The 26RFa (or 43RFa; currently known as QRFP43) protein is one of the most recently identified member of this peptide family. Studies showed that the sequence encoding 26RFa precursor is present and expressed in the brain region in all vertebrates^3^. In mammals, cleavage of the 26RFa precursor can generate two forms of this peptide: 26RFa and QRFP43, the latter being an N-terminal elongated form. Studies have demonstrated that both of them have the same biological activity^4,5^.

The results of research conducted in different species revealed that the highest levels of 26RFa/QRFP43 gene expression, as well as the localisation of neurons expressing 26RFa/QRFP43 were detected in the hypothalamic nuclei engaged in the regulation of the hypothalamo-pituitary–gonadal (HPG) axis, including the arcuate nucleus (ARC), paraventricular nucleus (PVN), and ventromedial hypothalamic nucleus (VMN)^3,4,6,7^. It is worth emphasising that the presence of GPR103 (26RFa/QRFP43 receptor) was also found in the same hypothalamic areas^8–10^. Furthermore, it has been demonstrated in rats that the GPR103 receptor subtype is located in the preoptic area (POA) and anterior hypothalamic area (AHA), which contains the main population of GnRH neurons in the hypothalamus^7,8^. The presence of 26RFa/QRFP43 and their receptor has also been confirmed in reproductive tissues, suggesting that it may additionally regulate reproductive processes directly at the gonadal level^11,12^.

The currently available information about the involvement of 26RFa/QRFP43 in the regulation of gonadotrophic axis activity is mainly derived from experiments performed in rodents. The results of a study conducted in cyclic and ovariectomised (OVX) rats demonstrated that intracerebroventricular (icv) injection of both 26RFa and QRFP43 increased luteinizing hormone (LH) and follicle stimulating hormone (FSH) levels in peripheral blood^13^. Those authors also showed that 26RFa stimulated the release of LH and FSH from the pituitary explants of male and female rats and enhanced GnRH-induced secretion of gonadotrophins in male rats^13^. A similar effect was observed in male rats, in which central administration of QRFP43 elevated LH and FSH levels. Moreover, QRFP43 was found in in vitro studies to induce GnRH release from hypothalamic explants, and pretreatment with GnRH receptor antagonist blocked the QRFP43-induced increase in plasma LH levels^14^. These findings collectively suggests that in rodents, 26RFa/QRFP43 may modulate the HPG axis by acting both at the hypothalamic level on GnRH neurons and at the pituitary level on gonadotrophe cells.

The available literature lacks concise information regarding the specific neural pathways through which 26RFa/QRFP43 may affect reproductive regulatory centres in the hypothalamus. It is not known whether 26RFa/QRFP43 acts directly on GnRH neurons, or indirectly, by altering the activity of neurons that make up the appetite regulatory centre or neurons forming the GnRH pulse generator (kisspeptin/neurokinin B/dynorphin neurons; as described in the literature^15–19^). There is also little information about the participation of 26RFa/QRFP43 in the regulation of gonadotrophic axis function in animals other than rodents.

The planned research focuses on the neuromodulatory effect of QRFP43 in the central nervous system (CNS) in the regulation of reproductive processes. To this end, we have selected Polish Merino sheep as the animal model utilised in our studies. Although 26RFa and QRFP43 can both bind and activate GPR103, QRFP43 exhibits stronger agonist activity in vivo^14^. For this reason, we decided to use this peptide in the present study.

In this study, we investigated the in vivo effect of icv QRFP43 infusion into the third ventricle (IIIv) of the sheep brain on the secretory activity of key gonadotrophic axis hormones that regulate reproductive processes. We examined mRNA and peptide expression levels of GnRH and KNDy in the hypothalamus and mRNA and protein expression levels of LHβ and FSHβ in the pituitary gland. Moreover, we investigated alterations in LH and FSH concentrations in the blood.

## Materials and methods

### Animals and experimental design

All procedures were conducted in accordance with the Code of Ethics of the World Medical Association (Declaration of Helsinki), as well as the EU Directive 2010/63/EU on animal experimentation. Approval for the study (no. WAW2/193/2019) was obtained from the Local Ethics Committee affiliated with Warsaw University of Life Sciences, in compliance with the Polish Law for the Animal Care and Use of January 21, 2005 and the Polish Law for Animal Protection (of September 16, 2011). Also presented experiment (design and all procedures) fulfil criteria of ARRIVE guidelines.

Forty-eight female Polish Merino sheep (42-week-old, average weight = 38.6 ± 3.5 kg) were used in the study. Animals were housed indoors under natural lighting conditions (52° N, 21° E) and fed a standard hay diet with commercial concentrates twice daily, according to the Polish Recommendations for Growing Sheep^20^. The feed was balanced in terms of energy and appropriate nutrients to ensure optimal development of the growing animals. Water and salt licks were available ad libitum. The reproductive status of each animal was assessed weekly (from the beginning of August) by determining plasma progesterone concentrations, as well as through post-mortem ovary examination (data not shown).

Stainless steel cannulas were implanted directly into IIIv of the brain^21^ under anaesthesia with atropinum sulfuricum (0.44 mg kg^−1^; Polfa, Warsaw, Poland), ketamine (400 mg per sheep; Vetoquinol Biowet, Gorzów, Poland), and dexmedetomidine (0.05 mg kg^−1^, Dexdomitor®; Orion Pharma, Turku, Finland). Permanent stainless-steel guide cannulas (ø = 0.8 mm) were inserted into the IIIv (coordinates of guide cannula implantation: antero-posterior position—31 mm, horizontal position—0.5 mm, mid-sagittal position—0.10 mm) according to Welento et al.^22^. The correct placement of the guide cannula was verified by the efflux of cerebrospinal fluid (CSF) upon removal of the guide tube stiletto, as well as by post-mortem inspection. After surgery, penicillin–streptomycin (0.1 mL kg^−1^; ScanVet, Gniezno, Poland) and tolfenamic acid (0.05 mL kg^−1^, Tolfine®; Vetoquinol Biowet, Gorzów, Poland) were administered by subcutaneous injection for four consecutive days, followed by a five-week recovery period. In all experimental animals, oestrus synchronisation was performed 21 days before icv infusion using Chronogest CR sponges (MSD Animal Health, UK), as described by Przybył et al.^23^. Animals entered the experiment on day 4 to 5 after ovulation, when a decrease in peripheral blood oestrogen levels is observed in sheep^24^.

The experiments were conducted from the end of October to the first week of December. Animals were randomly divided into three experimental groups: control group receiving an icv infusion of Ringer-Locke solution (artificial cerebrospinal fluid; 480 µL per day; n = 16), group 1 receiving icv infusion of QRFP43 (Phoenix Pharmaceuticals Inc., USA) at 10 μg per day (RFa10 group; n = 16), and group 2 receiving QRFP43 at 50 μg per day (RFa50 group; n = 16), both diluted in 480 µL of Ringer-Locke solution, at an infusion rate of 120 µL h^−1^. The doses of QRFP43 used in this experiment were selected based on preliminary research results (unpublished data).

During the experiment, 1 h prior to infusion, cannulas were introduced through the guide cannulas and locked in position with tips placed approximately 2.0–2.5 mm above the base of the brain; when the tips of the cannulas were in the IIIv, cerebrospinal fluid flowed into the infused cannulas. Subsequently, all sheep received four 50-min icv infusions at 30-min intervals, performed from 08:40 a.m. to 01:30 p.m. on three consecutive days^23,25^. The infusions were administered using a BAS Bee MD_1020 904 microinjection pump (Bioanalytica Systems Inc., West Lafayette, IN, USA), and to prevent a rapid increase in pressure during Ringer-Locke and QRFP43 infusions into the IIIv, the flow rate of the microinjection pump was set to 2 μL min^-1^. Blood samples has been collected from animals on Day 0 (before the infusion) and on Day 3 of infusion (from 08:00 a.m. to 02.00 p.m.) at 10 min. interval (36 samples were collected from each sheep on Day 0 as well as on Day 3).

Immediately after the last infusion, animals were weighed, anaesthetised intravenously using dexmedetomidine (0.05 mL kg^−1^, Dexdomitor®; Orion Pharma, Turku, Finland) and pentobarbital (80 ng kg^−1^, Morbital®; Vetoquinol Biowet, Poland), and then decapitated. Isolation of selected hypothalamic structures (area and depth of the cut) was performed in accordance with the stereotaxic coordinates defining the individual hypothalamic nuclei described in the anatomic atlas of the sheep hypothalamus^22^.

For molecular biological analysis (n = 8 + 8 + 8), isolation of the selected hypothalamic structures (area and depth of cuts) was performed according to the coordinates defining the location of individual hypothalamic nuclei described in the stereotaxic atlas of the sheep hypothalamus^22^. Four immunohistochemistry hypothalamic structures from eight sheep from each group (n = 8 + 8 + 8) were prepared as described by Przybył et al.^23^ and Szlis et al.^25^. Immediately after decapitation, brain from each sheep were perfused via both carotid arteries with 1000 ml 0.1 M phosphate buffered saline (PBS) and subsequently with 2000 mL 0.1 M PBS containing 4% paraformaldehyde (w/v). The hypothalami were dissected out ~ 20 min after the beginning of perfusion and fixed for a further 72 h by immersion in the same fixative. Next, they were formed in blocks of the same dimension and cutting face and the tissue was then washed with 0.01 M PBS, cryoprotected in a 20% sucrose solution in 0.1 M PBS for at least 2 days and stored at 4 °C. The hypothalamic blocks were frozen at -10 °C and cut on a cryostat in coronal planes at 30 µm sections.

### Real time RT-qPCR

Total mRNA from the POA and medial basal hypothalamus (MBH) was extracted using the NucleoSpin RNA/Protein kit (Macherey–Nagel GmbH & Co., Düren, Germany) according to the manufacturer’s instructions. The yield of isolated RNA was estimated spectrophotometrically (Nanodrop, NanoDrop Technologies, DE USA), and its integrity was evaluated electrophoretically by separation on a 1.5% agarose gel containing ethidium bromide. For complementary DNA (cDNA) synthesis, 1500 ng mL^−1^ mRNA from the selected hypothalamic regions in a total volume of 20 µL was reverse–transcribed using a TranScriba Kit (A&A Biotechnology, Gdynia, Poland) according to the manufacturer’s protocol. Species-specific primers for sheep (*Ovis aries*) determining the expression of housekeeping genes and genes of interest were designed using Primer3web, version 4.1.0 (The Whitehead Institute, Cambridge, MA; https://primer3.ut.ee/) and synthesised by Genomed (Warsaw, Poland). The primer sequences are listed in Table 1.

**Table 1:** The primer sequences used in the experiment.

Gene	Primer	Sequence (5'-3')	Product size (nt)	References
*gapdh*	Forward	AGAAGGCTGGGGCTCACT	134	^41,42^
	Reverse	GGCATTGCTGACAATCTTGA		
*gnrh*	Forward	GCCCTGGAGGAAAGAGAAAT	123	^43^
	Reverse	GAGGAGAATGGGACTGGTGA		
*gnrh-r*	Forward	TCTTTGCTGGACCACAGTTAT	150	^44^
	Reverse	GGCAGCTGAAGGTGAAAAAG		
*lhβ*	Forward	AGATGCTCCAGGGACTGCT	184	^24^
	Reverse	TGCTTCATGCTGAGGCAGTA		
*fshβ*	Forward	TATTGCTACACCCGGGACTT	131	^24^
	Reverse	TACAGGGAGTCTGCATGGTG		
*kiss*	Forward	GGATGAACGTGCTGCTT	106	^22^
	Reverse	CCTGTGGTTCTAGGATTCTC		
*pdyn*	Forward	CCAGTGCTCCTTGTGTGCTGT	216	^22^
	Reverse	GCCGGCTCACTGCTAGACTCT		
*nkb*	Forward	TTGGTGCGGTCTGTGAGGAGTCA	340	^22^
	Reverse	CAGGAGAGGAGTGCTCATTCCACA		

Real Time qPCR was performed using 5 × FIREPol EvaGreen qPCR Mix Plus (no ROX; Solis BioDyne, Tartu, Estonia) in a total volume of 15 µL containing 3 µL of Master Mix, 9 µL of RNase-free H_2_O, 2 × 0.5 µL of primers (0.5 mM), and 2 µL of cDNA template. Amplification was performed using a Rotor Gene 6000 thermocycler (Corbett Research, Mortlake, Australia) according to the protocol described by Przybył et al.^23^. Relative gene expression was calculated using the comparative quantitation option of the Rotor Gene 6000 software 1.7 (Qiagen GmbH, Hilden, Germany), as well as the Relative Expression Software Tool and the PCR efficiency correction algorithm developed by Pfaffl et al.^26,27^. The experiment used glyceraldehyde-3-phosphate dehydrogenase (GAPDH), β-actin (ACTB) and peptidylprolyl isomerase C (PPIC) as housekeeping genes and. GAPDH was selected using the Best-Keeper software (http://www.gene-quantification.de/bestkeeper.html) as the most optimal endogenous control to normalise gene expression. The results are presented as the relative expression of the target gene normalised to the housekeeping gene (GAPDH) with the relative gene expression for the group of sheep that received only Ringer-Locke solution infusion set to 1.0^25^.

### Immunohistochemical procedures

Hypothalamic sections were incubated for 12 days at 4 °C with primary GnRH antiserum (rabbit anti-GnRH ref. EPR24529-70; ABcam, Cambridge, UK), at a dilution of 1:800, or 8 days at 4 °C with primary Kiss antiserum (rabbit anti-Kiss 10 ref. 564; donated by Dr. Alain Caraty, INRA/CNRS/Universite Francois-Rabelais, Tours; described in Franceschini et al.^28^) at a dilution of 1:5000, in accordance with the procedure used in our laboratory^24^, with minor modification. After incubation with primary antibodies, the sections were incubated for 2 h at room temperature (~ 20 °C) with secondary antibody (peroxidase-conjugated sheep anti-rabbit Ig [H + L]; ABcam, Cambridge, UK) at a dilution of 1:400 in 0.1% normal lamb serum in 0.01 M PBS. The colour reaction was developed by incubating the sections with 0.05% 3′3-diaminobenzidine tetrahydrochloride chromogen (Sigma, St. Louis, MO, USA) and 0.001% hydrogen peroxide in 0.05 M Tris buffer. Selected hypothalamic material was also stained using the silver intensification method, as described by Liposits et al.^30^.

Peroxidase-conjugated antibodies were applied to identify pituitary cells containing LH- and FSH. The sections were incubated for 30 min in 2% pre-immune lamb serum, followed by 30 min in 0.1% hydrogen peroxide, both dissolved in 0.01 M PBS. Primary rabbit anti-sheep LHβ (dilution 1:800) and FSHβ (dilution 1:1400) antibodies were applied at 4 °C for 4 days, and the sections were subsequently rinsed in 0.01 M PBS and incubated with secondary antibody (sheep anti-rabbit IgG [H + L] conjugated with peroxidase; ABcam, UK) at a dilution of 1:800 for 2 h at room temperature in 0.1% normal lamb serum in 0.01 M PBS. The colourimetric reaction was initiated by incubating the sections with 0.05% 3′3-diaminobenzidine tetrahydrochloride chromogen (Sigma, St. Louis, MO, USA) and 0.001% hydrogen peroxide in 0.05 M Tris buffer.

To evaluate staining specificity, GnRH, Kiss, LH and FSH antisera were preabsorbed using synthetic GnRH (UCB, Belgium), Kiss10 (NeoMPS), LH (rLH32V02, NHPP, USA) and FSH (rFSH32V02, NHPP, USA), as described by Wójcik-Gładysz et al.^18^ and Szlis et al.^25^. No specific cell staining was observed in any of the above controls (data not shown).

### Image analyses

Histological analyses of pituitary sections were conducted using a Nikon type 104 projection microscope (Nikon Corporation, Tokyo, Japan). Images of immunostained sections were captured using a Panasonic KR222 camera (Matsushita Electric Industrial Co, Osaka, Japan) and displayed on a colour monitor. Pictures were adjusted for optimal contrast, converted to grey scale, fixed at the same brightness levels, and saved in a buffering system. Analyses were performed using a 10 × objective lens for GnRH perikarya in the POA as well as Kiss perikarya in ARC and 4 × objective lens for the nerve terminals in the ME as well as 40 × objective lens for pituitary. The parameter of area fraction, which indicates the percentage of stained elements in a delineated area, was used for immunoreactive (IR) GnRH perikarya in the POA, IR nerve terminals in the ME and IR LH and FSH in pituitary^23^.

Quantitative analysis was performed within the subareas of interest in each hypothalamus. This was performed by applying a threshold function to select a range of grey values that were optically identified as positively stained. All other values were referred to as unstained. Before taking measurements, the images were processed to subtract the background and remove artifacts. The threshold values used were different for the POA and ME, but constant for all measured images of one histochemical product. The frame size was kept constant for the duration of the image analysis. The area fractions of the immunoproduct in GnRH neurons were estimated in (i) every 8th section through the POA on both sides of the third ventricle of the brain (10 sections per animal, 2 fields of 0.3286 mm 2 measured in each section), and (ii) every 4^th^ section in the MBH in the delineated area containing the middle part of the ME (~ 20 sections per animal, total area of 100 mm^2^) for each hypothalamus). The area fractions of the LH and FSH immunoproduct were estimated in every 25 section per animal (total fields of 0.1308 mm^2^). The quantitative measurements taken from each section of the POA, ME and pituitary were averaged to obtain a mean from each area, for each animal^18^.

### LH and FSH radioimmunology

Plasma LH and FSH concentrations were determined according to the protocols previously described by Szlis et al.^25^ and Wójcik-Gładysz et al.^31,32^. The intra- and inter-assay coefficients of variation averaged 4.0% and 10.0% for LH and 3.3% and 11.3% for FSH, respectively. The mean concentrations of LH and FSH for individual animals were calculated from the area under the curve, which represents the sum of trapezoidal areas between the curve and the abscissa axis^25^.

### Statistical analysis

GraphPad Prism 5 software (GraphPad Software Inc., La Jolla, CA) was used for all statistical calculations. All data presented in the graphs represent means ± SEM for each group. Normality of data distribution was tested using the Shapiro–Wilk test. Statistical evaluations of the data were performed using the Kruskal–Wallis test with the post-hoc Dunn multiple comparisons test (for RIA and genes) or one-way ANOVA with the post-hoc Tuckey multiple comparisons test. Differences resulting in P ≤ 0.05 were considered statistically significant^25^.

### Ethical statement

All procedures were conducted in accordance with the Code of Ethics of the World Medical Association (Declaration of Helsinki), as well as the EU Directive 2010/63/EU on animal experimentation. Approval for the study (no. WAW2/193/2019) was obtained from the Local Ethics Committee affiliated with Warsaw University of Life Sciences, in compliance with the Polish Law for the Animal Care and Use of January 21, 2005 and the Polish Law for Animal Protection (of September 16, 2011). Also presented experiment (design and all procedures) fulfil criteria of ARRIVE guidelines.

## Results

### Expression of GnRH mRNA

**Figure 1:**
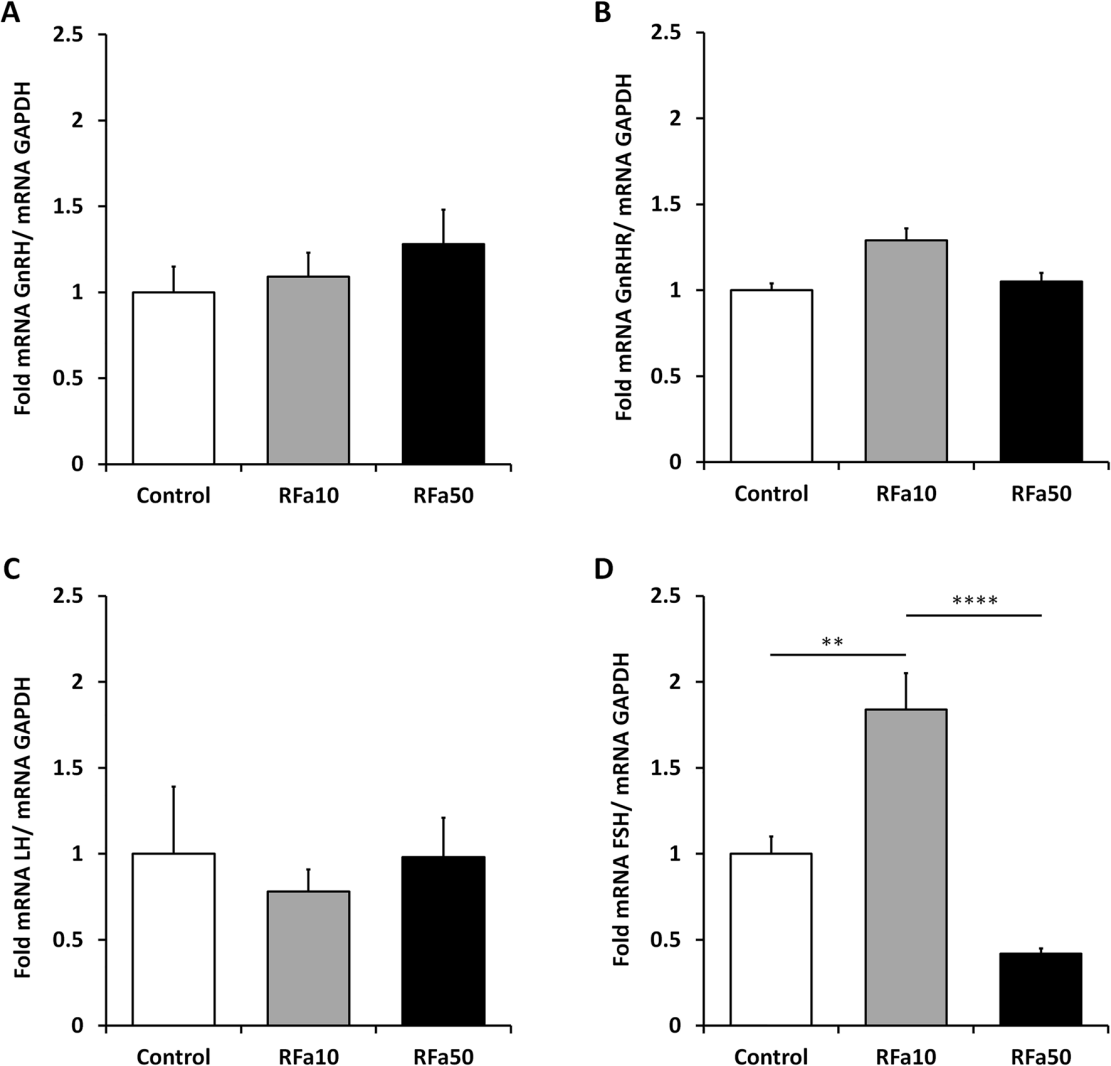
Expression of GnRH (**A**) mRNA in preoptic area (POA) and expression of GnRH-R (**B**), LHβ (**C**) and FSHβ (**D**) mRNA in pituitary. All values are relative to GAPDH mRNA. Data are means ± SEM, significantly different at *P < 0.05, **P < 0.01, ***P < 0.001, ****P < 0.00001.

Real Time qPCR analyses revealed the presence of GnRH mRNA transcript in the sheep POA in all experimental groups. However, there were no significant changes in GnRH mRNA expression in the sheep from any experimental group (Fig. 1A).

### Expression of Kiss, pDyn, and NKB mRNA

Real Time qPCR analyses showed that Kiss, NKB, and pDYN mRNA transcripts were present in the MBH of sheep from all experimental groups. Compared to control animals, Kiss mRNA expression was significantly (P ≤ 0.01) reduced in the RFa50 group (1.00-fold ± 0.11and 0.64-fold ± 0.01, respectively). Significant differences were neither observed between the RFa10 and controls group nor between the RFa10 and RFa50 groups.

**Figure 2:**
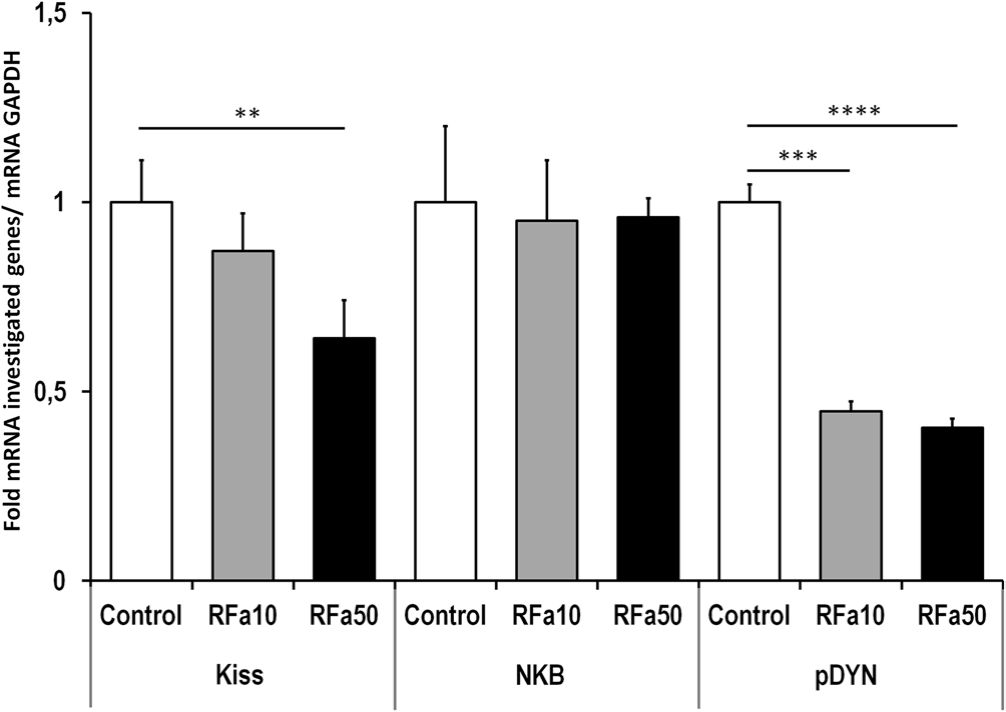
Expression of kisspeptin, preprodynorphin and neurokinin B mRNA in mediobasal hypothalamus. All values are relative to GAPDH mRNA. Data are means ± SEM, *P < 0.05, **P < 0.01, ***P < 0.001, ****P < 0.00001.

There were no significant changes in NKB mRNA expression in the sheep from any experimental group. However, pDYN mRNA expression was markedly (P ≤ 0.001 and P ≤ 0.0001, respectively) decreased in the RFa10 and RFa50 groups (0.45-fold ± 0.03 and 0.40-fold ± 0.02, respectively) compared to the control group (1.00-fold ± 0.05, respectively) of animals (Fig. 2).

### Expression of GnRH-R, LHβ, and FSHβ mRNA

At the pituitary level, QRFP43 was shown to alter only the level of FSHβ mRNA expression. An increase (1.84-fold ± 0.21, P ≤ 0.01) in FSHβ mRNA expression was observed in the RFa10 group compared to the control group. The expression of FSHβ mRNA was also significantly (0.42-fold ± 0.03, P ≤ 0.0001) lower in the RFa50 group than in the RFa10 group (Fig. 1D). No statistically significantly changes were observed in both GnRHR mRNA and LHβ mRNA expression in the pituitary in all groups of animals (Fig. 1B,C).

### GnRH immunoreactivity

Microscopic observations revealed that the location of immunoreactive GnRH material in the ME differed between the control group and both groups of sheep infused with QRFP43. In control animals, immunoreactive (IR) GnRH material was present in all three sections of the ME (anterior, medial and posterior ME), mainly in its external zone, on the entire width from its postero-anterior part to the pituitary stalk. In the QRFP43-infused groups of sheep, IR GnRH material was observed only in the medial and posterior sections of the ME, in its external zone with the highest accumulation in the postero-anterior part of the ME. No perikarya have been observed in the POA in any of the three groups of sheep.

**Figure 3:**
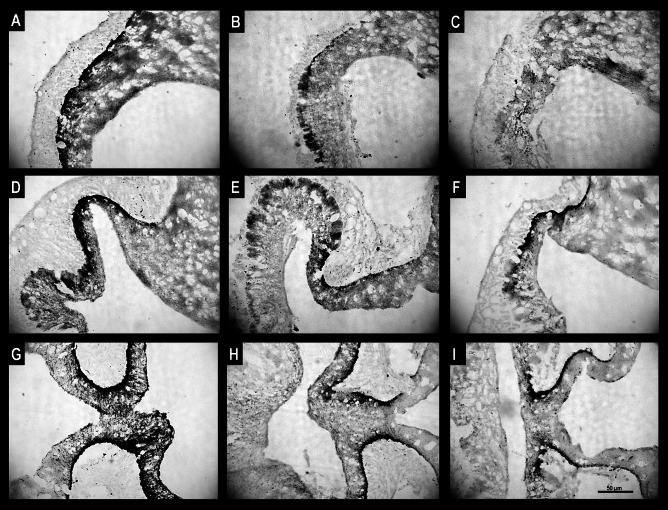
Population of immunoreactive GnRH nerve terminals in the anterior (**A**–**C**) medial (**D**–**F**) and posterior (**G**–**I**) part of the median eminence of representative sheep from the control group (**A**,**D**,**G**), RFa10 group (**B**,**E**,**H**) and RFa50 group (**C**,**F**,**I**). Scale bars: (**A**–**I**) 50 μm.

Furthermore, infusion of QRFP43 in both doses resulted in distinct changes in the amount of IR GnRH material in the ME. In control sheep, the immunoproduct located in nerve terminals of the ME was very abundant. In contrast, in QRFP43-infused sheep, the immunoproduct was relatively scarce throughout the entire width of the ME (Fig. 3). Those observation were supported by quantative analysis of image which demonstrated that in RFa10 and RFa50 groups the percentage of area exhibiting positive staining for GnRH in EM statistically (P < 0.001) decreased (2.73 ± 0.25% and 2.24 ± 0.14%, respectively) in comparison with the control group (6.65 ± 0.16%; Fig. 5A).

### Kisspeptin immunoreactivity

**Figure 4:**
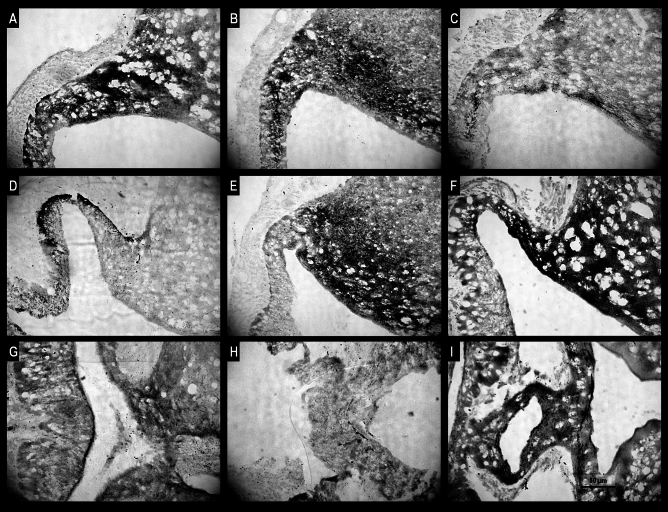
Population of immunoreactive kisspeptin perykaria and nerve terminals in the anterior (**A**–**C**) medial (**D**–**F**) and posterior (**G**–**I**) part of the median eminence of representative sheep from the control group (**A**,**D**,**G**), RFa10 group (**B**,**E**,**H**) and RFa50 group (**C**,**F**,**I**). Scale bars: (**A**–**I**) 50 μm.

Immunoreactive (IR) Kiss material was localised in all neuronal cell elements, including perikarya, fibres and terminals in the hypothalamic ARC and ME. In the control sheep, a significant portion of Kiss nerve terminals was immunoreactive in the anterior and medial but not in the posterior part of the ME (Fig. 4A,D,G). Furthermore, a few IR Kiss perikarya were noted in the ARC of the control sheep. Microscopic observations demonstrated that in QRFP43-infused groups of animals, IR Kiss terminals in all parts of the ME were sparse and weekly stained (Fig. 4B,C,E,F,H,I). In contrast to the control group of sheep, IR Kiss material was strongly stained in the entire ARC area (including perikarya and fibres) in both QRFP43-infused groups of animals (Fig. 4B,C,E,F,H,I).

**Figure 5:**
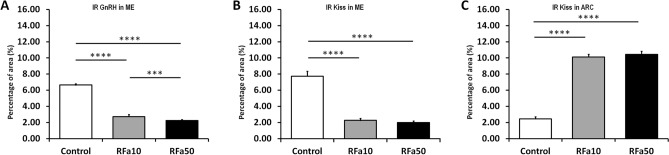
The percentage of total area of IR GnRH in median eminence (**A**), IR Kiss in median eminence (**B**) and IR Kiss in arcuate nucleus (**C**). Data are means ± standard error of measurement. Values significantly different at *P < 0.05, **P < 0.01, ***P < 0.001, ****P < 0.00001.

Those microscopic observation were supported by quantative analysis of image which demonstrated that in RFa10 and RFa50 groups the percentage of area exhibiting positive staining for Kiss in EM statistically (P < 0.001) decreased (2.26 ± 0.25% and 1.99 ± 0.20%, respectively) in comparison with the control group (7.73 ± 0.60%; Fig. 5B). Furthermore, the percentage of area exhibiting positive staining for Kiss in ARC statistically (P < 0.001) increased in RFa10 and RFa50 (10.12 ± 0.32% and 10.44 ± 0.38%, respectively) groups of sheep compared to control group of animals (2.45 ± 0.25%; Fig. 5C).

### LH and FSH immunoreactivity

**Figure 6:**
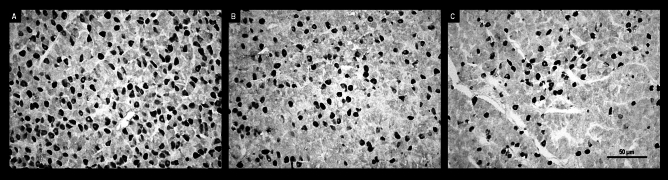
Populations of immunoreactive LH pituitary cells from control (**A**), RFa10 (**B**) and RFa50 (**C**) groups, scale bars: (**A**–**C**) 50 μm.

Microscopic observations showed visible differences in IR LH and IR FSH pituitary cells between the control group and both sheep groups administered QRFP43. The number of LH-positive stained cells and the intensity of the immunostaining were higher in control animals compared to the QRFP43-infused sheep. Moreover, a dose-depended decrease was observed in IR LH in pituitary cells after central administration of QRFP43 (Fig. 6). Those observation were supported by quantative analysis of image which demonstrated that in RFa10 and RFa50 groups the percentage of area exhibiting positive staining for LH in pituitary statistically (P < 0.001) decreased (3.48 ± 0.37% and 2.47 ± 0.29%, respectively) in comparison with the control group (6.55 ± 0.30%). Also, statistical (P < 0.001) decrease has been noted between RFa10 and RFa50 group of sheep (Fig. 8A).

**Figure 7:**
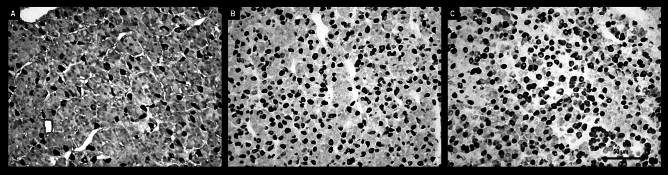
Populations of immunoreactive FSH pituitary cells from control (**A**), RFa10 (**B**) and RFa50 (**C**) groups, scale bars: (**A**–**C**) 50 μm.

**Figure 8:**
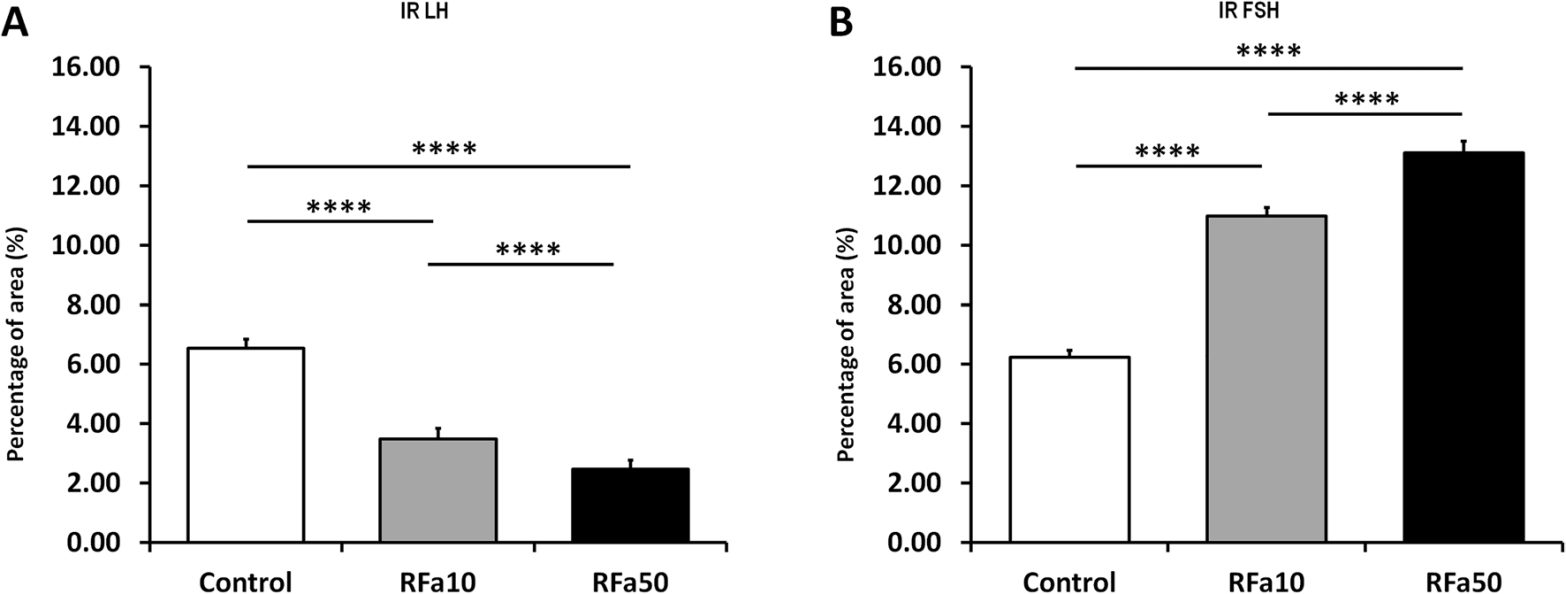
The percentage of total area of IR LH (**A**) and IR FSH (**B**) in pituitary. Data are means ± standard error of measurement. Values significantly different at *P < 0.05, **P < 0.01, ***P < 0.001, ****P < 0.00001.

In contrast to IR LH, QRFP43 increased the number of FSH-positive stained cells, as well as the intensity of the immunostaining in the RFa10 and RFa50 groups of sheep compared to control animals, and this effect was also dose-dependent (Fig. 7). Conducted observation were supported by quantitative analysis of image which demonstrated that in RFa10 and RFa50 groups the percentage of area exhibiting positive staining for FSH in pituitary statistically (P < 0.001) increased (10.99 ± 0.28% and 13.10 ± 0.25%, respectively) in comparison with the control group (6.23 ± 0.25%). Also, statistical (P < 0.001) increase has been noted between RFa10 and RFa50 group of sheep (Fig. 8B).

### Plasma LH and FSH concentrations and release pattern

**Figure 9:**
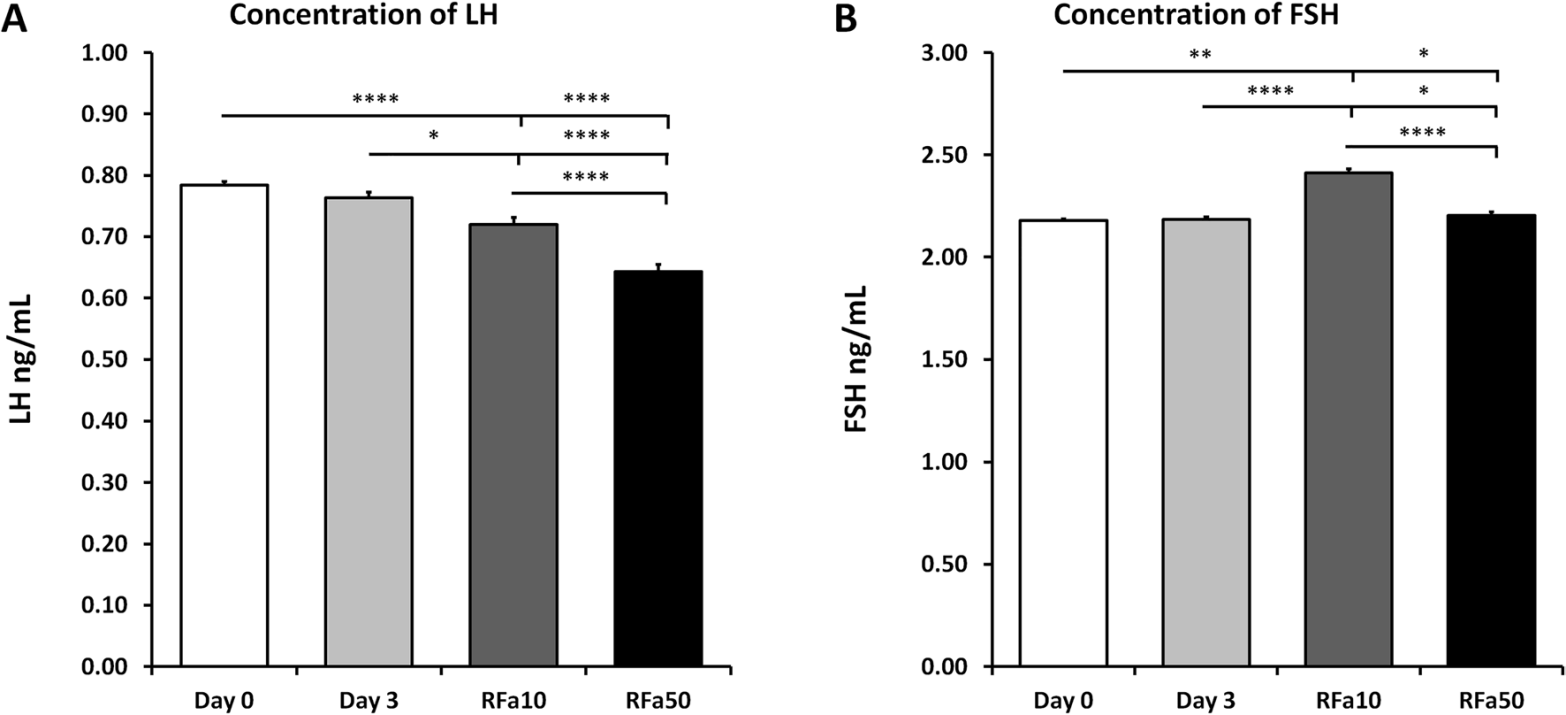
The concentration of luteinizing hormone (LH; **A**) and follicle-stimulating hormone (FSH; **B**) in blood plasma. Data are means ± standard error of measurement. Values significantly different at *P < 0.05, **P < 0.01, ***P < 0.001, ****P < 0.00001.

The basal mean plasma LH and FSH concentrations on Days 0 and 3 did not differ in the control group of sheep (0.78 ± 0.01 ng/mL and 0.76 ± 0.01 ng/mL, respectively) . However, the mean plasma LH concentrations decreased in both the RFa10 and RFa50 groups (P < 0.0001; 0.72 ± 0.01 ng/mL and P < 0.0001; 0.64 ± 0.01 ng/mL, respectively) compared to the control sheep from Day 0 (0.78 ± 0.01 ng/mL). Furthermore, significant changes were observed on Day 3 between the RFa10 and RFa50 groups (P < 0.01; 0.72 ± 0.01 ng/mL and P < 0.0001; 0.64 ± 0.01 ng/mL, respectively) in comparison to the control sheep (0.76 ± 0.01 ng/mL). The mean plasma LH concentration in the RFa10 group was significantly higher than in RFa50 group (P < 0.0001; 0.72 ± 0.01 ng/mL vs. 0.64 ± 0.01 ng/mL, respectively) (Fig. 9A).

An increase in mean FSH concentrations was observed in the RFa10 group compared to control sheep on Day 0 (P < 0.001; 2.41 ± 0.02 ng/mL vs. 2.18 ± 0.01 ng/mL), as well as between the RFa10 group and control sheep on Day 3 (P < 0.0001; 2.41 ± 0.02 ng/mL vs. 2.18 ± 0.01 ng/mL). In addition, a significant difference was recorded between the RFa50 group and the control groups on Day 0 and Day 3 (P < 0.01; 2.21 ± 0.02 ng/mL vs 2.18 ± 0.01 ng/mL and 2.18 ± 0.01 ng/mL, respectively). Significant differences were also found between the RFa10 and RFa50 animal groups (P < 0.0001; 2.41 ± 0.02 ng/mL vs 2.21 ± 0.02 ng/mL, respectively; Fig. 9B).

**Figure 10:**
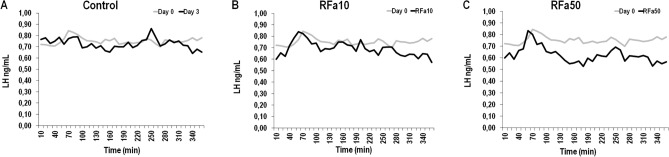
The release pattern of LH in representative individual sheep on day 0 and day 3 of infusion from control (**A**), RFa10 (**B**) and RFa50 (**C**) groups. RFa10—group of animals which received QRFP43 infusion at dose 10 μg/480 μl (n = 16); RFa50—group of animals which received QRFP43 infusion at dose 50 μg/480 μl (n = 16).

**Figure 11:**
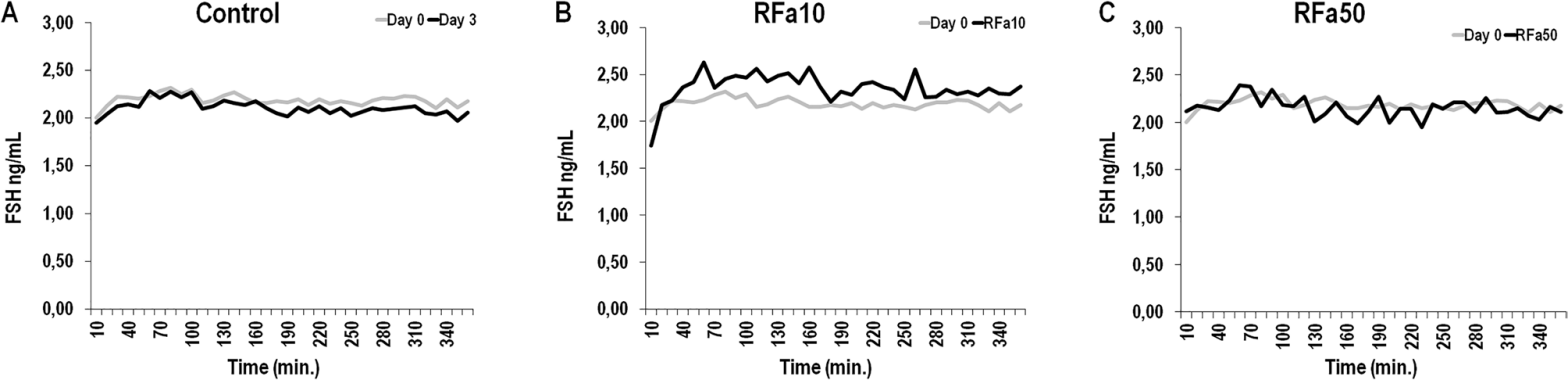
The release pattern of FSH in representative individual sheep on day 0 and day 3 of infusion from control (**A**), RFa10 (**B**) and RFa50 (**C**) groups. RFa10—group of animals which received QRFP43 infusion at dose 10 μg/480 μl (n = 16); RFa50—group of animals which received QRFP43 infusion at dose 50 μg/480 μl (n = 16).

The release pattern of LH and FSH in representative sheep from each animal group is presented in Figs. 10 and 11.

## Discussion

The results of our study indicated that centrally administered QRFP43 led to a decrease in GnRH protein expression in the hypothalamus. Moreover, changes in KNDy mRNA expression we observed in the MBH, as well as a reduction in Kiss protein expression in the ME after QRFP43 infusion. Central QRFP43 infusion inhibited the secretory activity of LH cells in the sheep pituitary. QRFP43 decreased the accumulation of LH protein in gonadotrophic cells, and as a result, peripheral blood LH levels were also reduced. At the same time, FSHβ mRNA expression remained unchanged, but protein accumulation in pituitary cells and blood FSH concentration increased after QRFP43 treatment. These differences in LH and FSH response patterns indicated that both gonadotrophe systems retained their specific sensitivity to central QRFP43 stimulation in ewes.

As mentioned earlier, RF-amide peptides are a family of related neuropeptides that regulate various physiological functions of the organism, including reproduction. Bioinformatic analysis has demonstrated that the amino acid sequence of this peptide exhibits high homology between individual vertebrate species, especially in the receptor domain. Available data on the action of RF-amide peptides demonstrate their differential effects on the HPG axis. The results of many studies have indicated that Kiss1 as well as GnIH acts as a regulator of GnRH release^33^. It is well known that Kiss1-synthesising neurons co-activate the GnRH pulse generator, and Kiss1 release is regulated in an auto- and paracrine manner by NKB and DYN. In all animals studied to date, Kiss1 has been shown to exert a very pronounced stimulatory effect, indicating that it is one of the most important peptides affecting GnRH release^34^. An increase in Kiss1 release from the nerve endings of KNDy neurons was shown to stimulate GnRH release into the pituitary portal circulation^35^. On the other hand, GnIH acts in an opposite manner by inhibiting the release of GnRH, which was first demonstrated in birds^36^. Subsequent investigations have shown that RFRP-3 is most likely the avian homologue of GnIH in the mammalian brain, which is responsible for modulating GnRH secretion^37,38^.

The present study found a decrease in pDYN mRNA expression in both QRFP43-infused groups of sheep, while no changes in NKB mRNA expression were recorded. This is a very surprising outcome, as in parallel, a downregulation of Kiss1 mRNA expression after QRFP43 treatment, with a significant reduction in the RFa50 group of animals was observed. Furthermore, image analyses of the hypothalamus have revealed unusual Kiss1 protein expression patterns after QRFP43 administration. The reduced localisation of IR Kiss1 material in ME nerve terminals and its strong accumulation in the ARC area suggested that the axonal transport of this neuropeptide was retained. These observations coincided with the observed reduction in the amount of IR GnRH material in the ME. The obtained data suggested that QRFP43 inhibited GnRH release from ME nerve terminals through its direct action on the secretory activity of Kiss1 neurons. This stays in contrast to other studies on RF26a and QRFP43 conducted in rats, which showed a stimulatory effect on GnRH release. One of these experiments involved male rats from which MBH explants were incubated with QRFP43 and CSF. RIA analyses of the post-incubation medium demonstrated that QRFP43 enhanced GnRH release from MBH explants^14^. Based on our observations, and the results of previous studies, it appears that the effect of QRFP43 may be species-specific and further studies should be performed concerning the relationships between QRFP43 and the GnRH pulse generator.

The findings of the present study suggest a correlation between pituitary and blood LH levels and the suppression of GnRH induced by QRFP43 treatment. At the pituitary level, immunohistochemical analyses revealed a reduced number of cells and IR LH material in the pituitary gonadotrophs of sheep treated with QRFP43. Additionally, QRFP43 infusion led to a decrease in LH concentrations in the peripheral blood of sheep. The opposite effect of RFamide peptides observed in the current trial was consistent with previous studies conducted in rats. In male rats, icv injection of QRFP43 increased both blood LH and FSH levels^13^, while other study in rats involving icv and peripheral administration of 26RFa and QRFP43 showed that 26RFa and QRFP43 administration stimulated LH secretion^13^.

In contrast to the demonstrated differences in LH levels, we observed a dose-dependent changes in FSH mRNA expression^13,14^. Exogenous icv infusion of QRFP43 resulted in a significant increase in FSH mRNA expression in the RFa10 group compared to control animals. Differences in FSH mRNA expression were also recorded between the two groups of sheep receiving QRFP43 infusion. Moreover, an increased number of IR FSH cells and IR material was observed in animals receiving QRFP43 infusion with a simultaneous increase in FSH concentration in the peripheral blood of sheep. The observed differences in LH and FSH stimulation could suggest a specific sensitivity of these hormones to central QRFP43 stimulation. It is well known that the regulation of FSH synthesis and release, unlike LH, is not fully dependent on the neuronal GnRH system and may respond differently to the hormones or neuropeptides tested. For instance, the administration of ghrelin into the third ventricle (IIIv) had no effect on LHβ mRNA expression, but it led to an increased accumulation of LH in the pituitary without altering the mean LH concentration in the blood. Interestingly, animals receiving icv infusions of ghrelin demonstrated a different response in the case of FSH. Ghrelin stimulation resulted in increased FSHβ mRNA expression and FSH accumulation in the pituitary, as well as an elevated levels of this hormone in the peripheral blood^31,32^. Similar findings were also reported in studies concerning the effect of obestatin on the gonadotrophic axis. Obestatin decreased LH concentration in pituitary cells, while simultaneously exhibiting a stimulatory effect on FSH accumulation^25^.

Subsequent studies on the mechanisms of RFRP-3 action in mammals have demonstrated that the effect of this peptide on HPG axis activity is complex and may vary depending on several factors. These factors include the species and sex of the animals, as well as their physiological condition and administration method of the compound. For example, a study conducted in OVX sheep receiving intravenous RFRP-3 infusion showed that it only reduced LH secretion without affecting blood FSH levels^39^. In contrast, another study also involving OVX sheep did not report any changes in plasma LH concentrations after intravenous RFRP-3 infusion^40^. Those findings suggest that the effect of RF-amide peptides are different for each family member and therefore it is necessary to conduct further research in this direction, especially those taking into account the demonstration of inter-species differences.

## Conclusion

In summary, the presented data showed that in sheep, QRFP43 suppressed the activity of the gonadotrophic axis at the hypothalamic level. The observed inhibition in GnRH expression and release from ME nerve terminals, which was associated with reduced Kiss peptide expression, resulted in a decrease in blood LH concentrations. Furthermore, it appears that QRFP43 can independently modulate FSH protein expression and release, possibly through other regulatory systems that require further investigations. Therefore, QRFP43 may serve as another neuromodulator of animal reproductive processes.

### Software used in experiments

1. Primer3web, version 4.1.0; The Whitehead Institute, Cambridge, MA; https://primer3.ut.ee/.
2. The Rotor Gene 6000 software 1.7 containing Relative Expression Software (Qiagen GmbH, Hilden, Germany); software provided by the manufacturer.
3. Best-Keeper software; http://www.gene-quantification.de/bestkeeper.html.
4. GraphPad Prism 5 (GraphPad Software Inc., La Jolla, CA); https://www.graphpad.com/features.

## Data Availability

The datasets used and/or analysed during the current study available from the corresponding author on reasonable request.

## Abbreviations

GnRH: Gonadotropin-releasing hormone
Kiss: Kisspeptin
GnIH: Gonadotropin-inhibitory hormone
HPG: Hypothalamo-pituitary–gonadal axis
ARC: Arcuate nucleus
PVN: Paraventricular nucleus
VMH: Ventromedial hypothalamic nucleus
POA: Preoptic area
AHA: Anterior hypothalamic area
OVX: Ovariectomised
icv: Intracerebroventricular
LH: Luteinizing hormone
FSH: Follicle stimulating hormone
CNS: Central nervous system
IIIv: Third ventricle of brain
MBH: Mediobasal hypothalamus
